# Exploring Online Crowdfunding for Cancer-Related Costs Among LGBTQ+ (Lesbian, Gay, Bisexual, Transgender, Queer, Plus) Cancer Survivors: Integration of Community-Engaged and Technology-Based Methodologies

**DOI:** 10.2196/51605

**Published:** 2023-10-30

**Authors:** Austin R Waters, Cindy Turner, Caleb W Easterly, Ida Tovar, Megan Mulvaney, Matt Poquadeck, Hailey Johnston, Lauren V Ghazal, Stephen A Rains, Kristin G Cloyes, Anne C Kirchhoff, Echo L Warner

**Affiliations:** 1 Department of Health Policy and Management Gillings School of Global Public Health University of North Carolina Chapel Hill, NC United States; 2 Cancer Control and Population Sciences Huntsman Cancer Institute at the University of Utah Salt Lake City, UT United States; 3 College of Nursing University of Utah Salt Lake City, UT United States; 4 Crowdfunding Cancer Costs LGBT Study Advisory Board Huntsman Cancer Institute at the University of Utah Salt Lake City, UT United States; 5 School of Public Health Indiana University Bloomington Bloomington, IN United States; 6 Wilmot Cancer Institute University of Rochester Medical Center Rochester, NY United States; 7 School of Nursing University of Rochester Rochester, NY United States; 8 Department of Communication University of Arizona Tucson, AZ United States; 9 School of Nursing Oregon Health & Science University Portland, OR United States; 10 Department of Pediatrics University of Utah School of Medicine Salt Lake City, UT United States

**Keywords:** community-engaged, LGBT, SGM, financial burden, crowdfunding, sexual monitory, sexual minorities, crowdfund, fund, funding, fundraising, fundraise, engagement, finance, financial, campaign, campaigns, web scraping, cancer, oncology, participatory, dictionary, dictionary, term, terms, terminology, terminologies, classification, underrepresented, equity, inequity, inequities, cost, costs

## Abstract

**Background:**

Cancer survivors frequently experience cancer-related financial burdens. The extent to which Lesbian, Gay, Bisexual, Transgender, Queer, Plus (LGBTQ+) populations experience cancer-related cost-coping behaviors such as crowdfunding is largely unknown, owing to a lack of sexual orientation and gender identity data collection and social stigma. Web-scraping has previously been used to evaluate inequities in online crowdfunding, but these methods alone do not adequately engage populations facing inequities.

**Objective:**

We describe the methodological process of integrating technology-based and community-engaged methods to explore the financial burden of cancer among LGBTQ+ individuals via online crowdfunding.

**Methods:**

To center the LGBTQ+ community, we followed community engagement guidelines by forming a study advisory board (SAB) of LGBTQ+ cancer survivors, caregivers, and professionals who were involved in every step of the research. SAB member engagement was tracked through quarterly SAB meeting attendance and an engagement survey. We then used web-scraping methods to extract a data set of online crowdfunding campaigns. The study team followed an integrated technology-based and community-engaged process to develop and refine term dictionaries for analyses. Term dictionaries were developed and refined in order to identify crowdfunding campaigns that were cancer- and LGBTQ+-related.

**Results:**

Advisory board engagement was high according to metrics of meeting attendance, meeting participation, and anonymous board feedback. In collaboration with the SAB, the term dictionaries were iteratively edited and refined. The LGBTQ+ term dictionary was developed by the study team, while the cancer term dictionary was refined from an existing dictionary. The advisory board and analytic team members manually coded against the term dictionary and performed quality checks until high confidence in correct classification was achieved using pairwise agreement. Through each phase of manual coding and quality checks, the advisory board identified more misclassified campaigns than the analytic team alone. When refining the LGBTQ+ term dictionary, the analytic team identified 11.8% misclassification while the SAB identified 20.7% misclassification. Once each term dictionary was finalized, the LGBTQ+ term dictionary resulted in a 95% pairwise agreement, while the cancer term dictionary resulted in an 89.2% pairwise agreement.

**Conclusions:**

The classification tools developed by integrating community-engaged and technology-based methods were more accurate because of the equity-based approach of centering LGBTQ+ voices and their lived experiences. This exemplar suggests integrating community-engaged and technology-based methods to study inequities is highly feasible and has applications beyond LGBTQ+ financial burden research.

## Introduction

Lesbian, Gay, Bisexual, Transgender, Queer, Plus (LGBTQ+) populations, which represent at least 7% of all US citizens [1], experience greater economic instability than their non-LGBTQ+ counterparts, including being more likely to live below the poverty line (24.6% vs 12.1%) and more likely to experience substantial identity-related employment discrimination [2-5]. LGBTQ+ people also experience higher rates of several of the most common cancer types and disproportionate cancer-related burdens [6,7]. Emerging literature suggests LGBTQ+ cancer survivors may be at an elevated risk for cancer-related financial burden [8]. Financial burden among LGBTQ+ cancer survivors may be further exacerbated by anti-LGBTQ+ bias, discrimination, and stigma including inadequate familial social and financial support due to rejection of an LGBTQ+ identity [8]. However, national surveys and health systems have only recently begun to collect sexual orientation and gender identity data, limiting researchers’ ability to study LGBTQ+ financial burden disparities [9,10].

At the same time, the volume of health-related information available online offers considerable opportunities to researchers adopting computational social science approaches [11]. In social media spaces and other online environments, LGBTQ+ identity is commonly disclosed through gendered language (eg, they/them) and self-disclosure when describing oneself [12]. Examples include posts on social media platforms and narratives included in crowdfunding campaigns. The latter is especially relevant for studies of medical financial burden since cancer survivors often use online crowdfunding for financial support and coping with cancer-related financial burden [13,14]. Thus, textual linguistic processing may offer an alternative mechanism to explore LGBTQ+ financial burden inequities.

Prior research into inequities in crowdfunding has often used web-scraping and machine learning methods to assemble and analyze data sets of health-related crowdfunding campaigns [15-18]. However, the use of machine learning to assist in identifying patients with stigmatized identities, such as LGBTQ+ identity, is potentially problematic as it may infuse the biases of the researchers into identification and analyses. These biases have a variety of potential consequences including findings that are not representative of the population of interest, or in the context of clinical decision-making tools, misdiagnoses of already vulnerable populations [19]. Such approaches may be problematic due to the LGBTQ+ population’s long history of stigmatization, exclusion, and discrimination inside and outside of the health care setting—anti-LGBTQ+ legislation and attitudes are embedded in US society and thus within researchers conducting big data analyses [20]. At the same time, the existing research focused on LGBTQ+ inequities in crowdfunding outside of the cancer context often do not use machine learning methods and instead use the search function of the crowdfunding website to identify LGBTQ+ campaigns [21,22]. Use of the search function may result in findings that are of unknown representativeness of LGBTQ+ campaigns on crowdfunding sites.

More accurate and reliable methodological approaches are needed to study LGBTQ+ inequities in the context of historical and current anti-LGBTQ+ attitudes and beliefs. Current literature suggests that LGBTQ+ research should shift from studying LGBTQ+ disparities to creating co-owned engaged research [23]. Thus, an equity-based methodological approach, wherein community members are included in the design, planning, implementation, analysis, and interpretation of results, is needed to explore LGBTQ+ inequities in cancer-related crowdfunding. The primary aim of this paper is to describe the process by which we integrated community-engaged and technology-based methods to explore inequities in crowdfunding for cancer-related costs between LGBTQ+ and non-LGBTQ+ cancer survivors—the Crowdfunding Cancer-related Costs among LGBTQ+ cancer survivors (C3 LGBT) study. The methods section includes descriptions of community-engaged research and web-scraping methodological approaches used to collect data. The results section includes descriptions of the integration of community-engaged and technology-based methods and the resulting identification tools and data set. Our results describe and explain how to integrate community-engaged and technology-based methods in research. While our study design was implemented for an LGBTQ+ and cancer-related topic, these examples on how to center equity in technology-based methods can be applied to a variety of existing outcomes and are intended to guide future researchers who wish to incorporate these unique methodological approaches.

## Methods

### Community-Engaged Research Methods

Community-engaged research is defined as a collaborative approach to research that includes the population being studied as informants in the development and execution of a research project [24]. Community involvement can include a variety of voices from the population of interest including leaders from relevant organizations as well as individual community members. Community-engaged research is positioned in epistemological paradigms outside of traditional positivism in which researchers assume that there is a universal truth to be discovered [25,26]. Rather, community-engaged methods promote colearning between the researchers and the community in a constructivist approach. Hallmarks of community-engaged research include but are not limited to building on strengths within the community, reciprocal mutually beneficial partnerships, cyclical processes, and engagement throughout the study and beyond [27]. Current community-engaged research methods exist on a continuum from community-informed research (ie, influenced by the community but no community involvement) to community-driven or led research (ie, support the community in conducting research) [28].

To center the LGBTQ+ community in the C3 LGBT study, we convened an LGBTQ+ study advisory board (SAB), with whom we developed and refined methods to scrape crowdfunding campaigns and accurately categorize them as LGBTQ+ and as cancer related. The goal of the SAB was to cocreate knowledge about LGBTQ+ cancer crowdfunding with the C3 LGBT analytic team by meeting to develop and refine study methods and participate in analyses. Individuals were eligible for the SAB if they (1) identified as LGBTQ+ and had a prior cancer diagnosis or cancer caregiving experience or (2) were clinical professionals working with the LGBTQ+ community. Recruitment, led by ARW and CT, included emailing a flyer with information about the SAB and study to professional contacts in LGBTQ+ research, existing cohorts of prior research participants who identified as LGBTQ+, and referrals from the Huntsman Cancer Institute at the University of Utah. Prior to recruitment, the C3 LGBT analytic team met to outline the role of SAB members in the study. Each SAB member would be expected to participate digitally via Zoom (Zoom Technologies, Inc) in at minimum 4 board meetings (60 minutes each) over the following year, receiving a US $200 per person honorarium for their time.

SAB members (n=8) worked with the C3 LGBT analytic team to further delineate their role and level of engagement with the outlined 4 meetings as the minimum level of engagement. That is, if board members were particularly interested in specific components or subprojects (eg, publishing or conference presenting), they were encouraged to discuss those ideas with the C3 LGBT analytic team and other SAB members. The SAB’s engagement was measured by (1) tracking attendance in regularly scheduled meetings and additional voluntary meetings, (2) asking for feedback about engagement during meetings via an anonymous poll, and (3) sending an individual-level survey prior to the final SAB meeting that assessed each member’s desired level of engagement with the proposed activities and provided an open space for feedback.

### Web-Scraping Methods

We assembled a data set of all active US-based medical campaigns hosted on GoFundMe, a large crowdfunding platform, and then used term detection to classify cancer-related campaigns benefitting LGBTQ+ individuals (discussed later). First, we accessed a list of all URLs that the platform makes available to search engines (the sitemap.xml). We downloaded the static HTML from each URL and, using the Beautiful Soup 4 Python library, extracted the campaign title, creation date, campaign category (medical vs other), campaign status (active vs inactive), donation amount, number of donors, organizer’s location, and the campaign description provided by the creator. Information that is not available in the static HTML, such as fundraising updates and donor comments, could not be captured using this method. We identified the campaign language using the langdetect library and excluded campaigns without a campaign description in English while retaining campaigns with descriptions in English and another language. Overall, 2,208,418 URLs were present in the sitemap.xml; of which 494,242 were active US medical campaigns written in English. Campaigns are determined to be medical campaigns by the user when they create the crowdfunding campaign. The sitemap.xml was accessed on November 14, 2022, and scraping was performed between November 14 and 22, 2022. All web scraping and data extraction were conducted in Python 3 (Python Software Foundation) [29].

### Composition and Positionality of the Study Team

The C3 LGBT study team consisted of 3 subgroups including the SAB (MM, MP, HJ, LVG, and others listed in acknowledgments), a team of faculty collaborators with content and conceptual expertise (SAR, KGC, and ACK), and the analytic team (ARW, CT, CWE, IT, and ELW). As part of the analytic process (July 2022-June 2023), the study team took time to reflect on their positionality or how individuals are influenced by their world views and the social positions they adopt, in relation to the C3 LGBT study and LGBTQ+ population. To guide this process, everyone who worked on the study was prompted to think about their positionality through 3 mechanisms including locating themselves in relation to the subject, locating themselves in relation to the participants, and locating themselves in the context of the research process [30]. Each individual wrote their positionality statement; keywords and phrases are displayed as a word cloud in Multimedia Appendix 1.

The study team held a variety of intersectional identities that informed the way that they approached the C3 LGBT study. Researchers on the analytic team and faculty collaborators were located in Utah, Arizona, and North Carolina. The analytic team and faculty collaborators held identities that ranged from completely removed from the LGBTQ+ community to identifying as a part of the LGBTQ+ community. Lead author and analyst, ARW, identifies as part of the LGBTQ+ community and has experience as a caregiver for chosen and blood family with serious illnesses including cancer. Other analytic team members did not identify as part of the LGBTQ+ community but shared familial ties to the community and other perspectives. Faculty collaborator KGC identifies as a part of the LGBTQ+ community, has close family members who are LGBTQ+, and has chronic and serious illness caregiving experiences with both chosen and blood family.

SAB board members nearly all identified as part of the LGBTQ+ community and included cancer survivors who lived across the United States (ie, Utah, Michigan, and New York). SAB members shared how their cancer intersected with their LGBTQ+ identity with 1 SAB member even sharing that they chose not to disclose their identity in their crowdfunding campaign during their treatment due to fear of anti-LGBTQ+ attitudes impacting their ability to raise funds. Reflecting on how researchers’ and community members’ frames of reference, epistemological points of view, and lenses influence research is crucial. Reflections from the study team highlighted discrimination within the LGBTQ+ community, societally held anti-LGBTQ+ attitudes, deep ties to cancer and caregiving as well as the complexity of identity—all of which inform how the study team approached the C3 LGBT study. While not common in quantitative research, reflexivity and positionality are important in analyzing and interpreting big data [31].

### Ethical Considerations

This study was considered exempt from ethics approval by the University of Utah’s institutional review board as it only involves publicly available data (IRB#00154744). Data were not anonymous nor deidentified as all data are actively available on GoFundMe and web-scraped to create this data set. SAB members are considered to be study team members not as study participants.

## Results

### Integration of Community-Engaged and Technology-Based Methods

**Figure 1:**
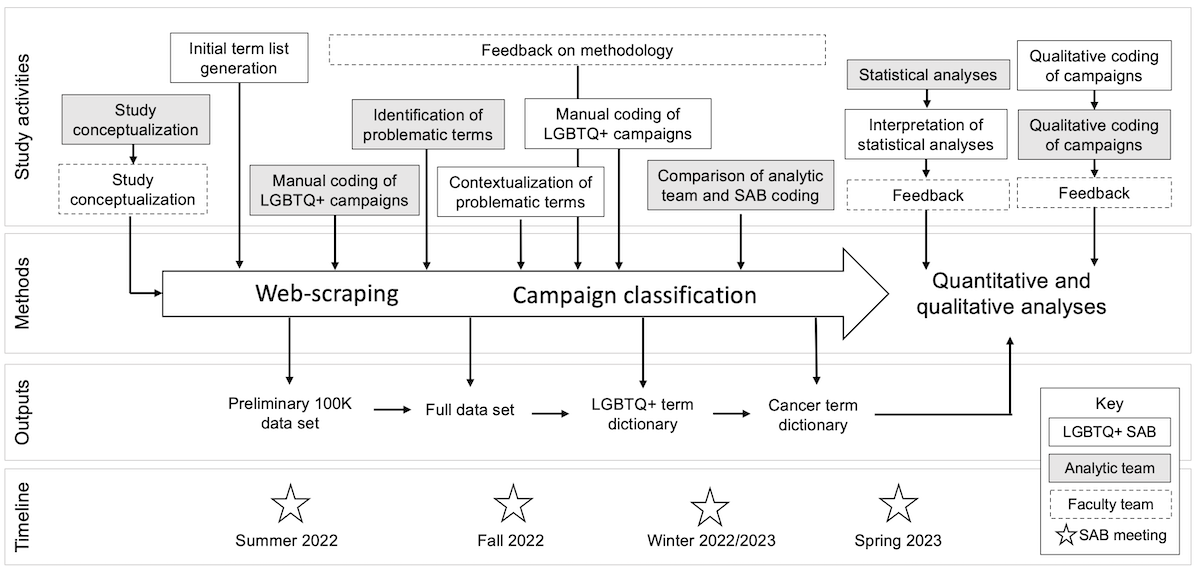
Study design of the Crowdfunding Cancer Costs LGBT study: integration of community-engaged and technology-based methods. LGTBQ+: Lesbian, Gay, Bisexual, Transgender, Queer, Plus; SAB: study advisory board.

True to the principles of community-engaged research, the C3 LGBT SAB was engaged during every step of the study. Across the 4 SAB meetings, none of the 8 SAB members dropped out, 4 SAB members did not miss any meetings, and 4 missed 1 meeting. All SAB members took part in an online engagement survey to express interest in additional participation in study activities in addition to SAB meetings, which included opportunities to perform qualitative coding, review manuscripts, and participate in manuscript dissemination—4 SAB members opted into additional activities. The SAB was also heavily involved in the creation, refinement, and testing of the term lists used to categorize crowdfunding campaigns as LGBTQ+ and as cancer related (Figure 1). The first iteration of the cancer-related term list was previously published by Silver et al [17], while the first iteration of the LGBTQ+ term list was developed by the analytic team. The SAB then provided feedback by adding and removing terms from each term list, focusing primarily on the LGBTQ+ term list. The term lists were then applied to the first small-batch scrape of 100,000 campaigns. The campaigns were filtered down to LGBTQ+ cancer campaigns as identified by the term lists. The analytic team and the SAB, independently and without knowledge of the term list assignment, manually coded each campaign identified as LGBTQ+ and cancer to test the accuracy of the term lists and the reliability of the analytic team and SAB. Coding done by the SAB was considered the gold standard for this analysis. Pairwise percent agreement was calculated between the term list categorization and SAB categorization as well as between the term list categorization and analytic team categorization. The refined term list including words to be quality checked was applied to a subset of the final data set, and pairwise percent agreement was calculated. The final term lists were then applied to the full campaign data set.

### Term Dictionary and Classification of LGBTQ+ Campaigns

From the first small batch scrape, a total of 93 LGBTQ+ campaigns were randomly selected from 33,478 cancer-related campaigns identified using the initial LGBTQ+ term list. After manual coding of the 93 campaigns by the analytic team, the analytic team determined that the search results yielded by the term list had correctly identified LGBTQ+ campaigns 88.2% of the time and misclassified them 11.8%. To ensure the analytic team coding was representative of LGBTQ+ community members’ interpretations, the SAB refined the term list in small breakout group discussions, and the term list was then reapplied to a small batch scrape with 87 LGBTQ+ campaigns. Manual coding by the SAB of 87 campaigns revealed a percent agreement of 79.3%, meaning that 20.7% of campaigns were identified by the SAB as being misclassified by the term list.

Reasons for misclassification were identified and included the use of the LGBTQ+ term list word “trans” used in medical terminology (eg, trans-metatarsal and trans-abdominal) as well as the LGBTQ+ term list word “gay” commonly occurring as a legal first or last name. Such terms were added to the term list that needed a manual quality check. Some LGBTQ+ term list words were also identified as commonly causing misclassification but did not discretely identify LGBTQ+ campaigns (ie, other words on the LGBTQ+ term list already identified the campaign as LGBTQ+ without the inclusion of such problematic terms) including “transitioning” and “fluid.” Such words were removed from the LGBTQ+ term list. Once the term list was finalized, manual coding of 100 LGBTQ+ classified campaigns by the analytic team revealed a final percent agreement with the LGBTQ+ term list of 95%. The iterations of the LGBTQ+ term list can be found in Textbox 1.

**Iterations of the lesbian, gay, bisexual, transgender, queer term list.**

Initial LGBTQ+ term list: 2-spirit, 2 spirit, 2S, Ace, AFAB, Agender, AMAB, Aromantic, Asexual, Assigned female at birth, Assigned male at birth, Bigender, Bisexual, Bottom surg, Demi, Drag k, Drag p, Drag q, Dyke, Dysphoria, Enby, Ey/, Fag, Femme, Fluid, Fruity, FTM, Gay, Gender-aff, Gender aff, Gender confirmation, Gender dysphoria, Gender euphoria, Gender f, Gender non, Gender queer, Gender transition, Genderf, Genderqueer, GNC, Her girlfriend, Her wife, His boyfriend, His husband, HRT, Intersex, Lesbian, LGBT, Masc, MTF, Mx., NB, Ne/, Non-binary, Nonbinary, Omnigender, Pansexual, Partner, Phalloplasty, Poly, QTPOC, Queer, Same-gender loving, Sex reassignment, Sexual and gender minority, SGM, They/, Top surgery, Trans, Transgender, Transitioning, Transsexual, Two-spirit, Two spirit, Vaginoplasty, Ve/, Xe/, Zie/

- Eliminated terms: Ace, Demi, Dyke, Femme, Fluid, Fruity, GNC, HRT, Masc, NB, Partner, Poly, Transitioning
- Terms to quality check: Gay, Trans
- Words with 0 hits: 2-spirit, 2 spirit, Agender, Aromantic, Bigender, Drag k, Enby, Ey/, Fag, Gender queer, Genderf, Intersex, MTF, Omnigender, Pansexual, Phalloplasty, Same-gender loving, Sexual and gender minority, SGM, Transsexual, Vaginoplasty, Xe/, Zie/
- Final term list: AFAB, AMAB, Asexual, Assigned female at birth, Assigned male at birth, Bisexual, Bottom surg, Drag p, Drag q, Dysphoria, FTM, Gay, Gender-aff, Gender aff, Gender confirmation, Gender dysphoria, Gender euphoria, Gender f, Gender non, Gender transition, Genderqueer, Her girlfriend, Her wife, His boyfriend, His husband, Lesbian, LGBT, Mx., Ne/, Non-binary, Nonbinary, QTPOC, Queer

### Term Dictionary and Classification of Cancer Campaigns

Of the small batch scrape, the same 93 campaigns were manually coded by the analytic team to identify agreement with the cancer term list. This additional check was performed to assess the accuracy of the cancer term list and refine it if needed. The analytic team revealed an 89.2% agreement with the cancer term list—10.8% misclassification. The SAB board then manually coded an additional randomly selected 89 cancer campaigns from the small batch scrape with 68.5% agreement. The analytic team and SAB agreed that many misclassified campaigns were comparing other diseases to cancer or tangentially mentioning a family member’s cancer. Specific treatment words such as “chemo” and “mastectomy” were identified by the SAB and analytic team to be driving misclassification as they were used in the context of other diseases. Such words were excluded from the term list after the SAB coding. Once the term list was finalized, manual coding of 93 campaigns by the analytic team revealed a percent agreement with the cancer term list of 89.2%. The iterations of the cancer term list can be found in Textbox 2.

**Iterations of the cancer term list.**

Cancer term list: adenocarcinoma, astrocytoma, cáncer, carcinoid, carcinoma, chemo, chemotherap, clear cell, desmoplastic, ductal carcinoma, ductile carcinoma, ependymoma, glioblastoma, histiocytosis, immuno therap, immunotherap, langerhans, leukemia, luekemia, lukemia germ cell tumor, lumpectomy, lymphoma, malignan, mastectomy, medulloblastoma, melanoma, myeloma, myloma, neruoblastoma, neurblastoma, neuroblastoma, neuroendocrine tumor, non-hodgkins lymphoma, non hodgkins lymphoma nonhodgkins lymphoma, nueroblastoma, nuroblastoma, oligodendroglioma, radiation therap, radiotherap, renal cell, retinoblastoma, rhabdomyosarcoma rhabdomyosaroma, sarcoma, seminoma, squamous cell, thymoma, wilm's tumor, wilms tumor

- Eliminated terms: chemo, chemotherap, immuno therap, immunotherap, mastectomy, radiation therap, radiotherap
- Final term list: adenocarcinoma, astrocytoma, cáncer, carcinoid, carcinoma, clear cell, desmoplastic, ductal carcinoma, ductile carcinoma, ependymoma, glioblastoma, histiocytosis, langerhans, leukemia, luekemia, lukemia germ cell tumor, lumpectomy, lymphoma, malignan, medulloblastoma, melanoma, myeloma, myloma, neruoblastoma, neurblastoma, neuroblastoma, neuroendocrine tumor, non-hodgkins lymphoma, non hodgkins lymphoma, nonhodgkins lymphoma, nueroblastoma, nuroblastoma, oligodendroglioma, renal cell, retinoblastoma, rhabdomyosarcoma, rhabdomyosaroma, sarcoma, seminoma, squamous cell, thymoma, wilm's tumor, wilms tumor

## Discussion

### Principal Findings

We sought to design a study combining community-engaged and technology-based methods to center the LGBTQ+ community and explore inequities that are unable to be assessed due to limited sexual orientation and gender identity data collection. The identification of LGBTQ+ and cancer-related crowdfunding campaigns was more accurate than it would have been otherwise when pairing community-engaged research methods with technology-based methods. For example, a principal observation made during the refinement of the LGBTQ+ and cancer term dictionaries for use on the GoFundMe data set was that the SAB consistently identified more misclassified campaigns (ie, campaigns that were automatically coded as LGBTQ+ but should not have been or vice versa) than the analytic team. Similarly, the SAB also expanded on the original LGBTQ+ term list that the analytic team developed. Taken together, these 2 results demonstrate the increased rigor of combining community-engaged study methods with technology-based approaches. Increased rigor may contribute to successful community engagement throughout the development and refinement of the 2 term dictionaries. The SAB regularly contributed justifications for adding and excluding terms based on their lived experiences within the LGBTQ+ and cancer communities. Not only did each member of the SAB importantly contribute their individual experiences and knowledge but the structure of the SAB (ie, quarterly meetings) allowed for members to share and cocreate new knowledge with the analytic team in real time.

Importantly, it was only by working together that the analytic team and SAB were able to produce an LGBTQ+ term dictionary with a pairwise agreement of 95%. This finding highlights the importance of centering the LGBTQ+ community in research involving LGBTQ+ cancer survivor outcomes, even if the chosen methodology may seem to not align with community-engaged equity-based methods, such as web-scraping and multivariate modeling. The integration of the SAB minimized the potential for misclassification and therefore minimized the bias of our future quantitative findings. Further, adequately engaging LGBTQ+ community members in technology-based methods confront the normalization of anti-LGBTQ+ attitudes, which can be seen in an unprecedented number of anti-LGBTQ+ bills in the past few years [32]. Avoiding algorithmic biases that mirror institutional biases (eg, racism) via equity-based methods is a growing priority in modern society [33]. There are a variety of potential negative implications when equity-based methods are not integrated into research protocols and cause bias in studies like the C3 LGBT study. The existing literature on the financial burden experienced by LGBTQ+ cancer survivors is sparse, with only a few studies that have directly assessed financial burden and none, to our knowledge, have assessed LGBTQ+ inequities in crowdfunding [8,34]. If the original term dictionary generated by the analytic team alone were used to identify LGBTQ+ campaigns, findings would have been inaccurate and would have had the potential to move financial burden research among LGBTQ+ survivors in the wrong direction.

Furthermore, these results can be contextualized in community-engaged research theory, which emphasizes principles of “connected knowing,” which is grounded in experiences, context, and relativism as opposed to “separate knowing,” which emphasizes logic, deduction, and absolute truth [35]. Approaching LGBTQ+ cancer research from a connected knowing lens is one way to potentially ameliorate stigma and discrimination experienced within this community by shifting away from traditional objectivist methods of deductively creating knowledge [36]. For this study, using a connected knowing lens allowed for necessary interpretation by SAB members to elucidate the inherent nuance found in our data set, thus addressing limitations created by using web-scraping methods alone.

We designed this study in alignment with several published recommendations for conducting research with LGBTQ+ cancer populations, which include cultivating non-cishetereonormative spaces typically found in health care research settings, prioritizing mutually beneficial relationships, and implementing sustainable interventions [23,37,38]. We aligned with these guidelines by centering LGBTQ+ voices from the SAB at every step of the research, encouraging SAB members to choose the activities that would be most advantageous for them, and developing a data set and term dictionaries that can be used for future LGBTQ+ research. Importantly, the SAB was compensated for their time and free to choose their level of engagement. It is possible that the high level of SAB engagement can be explained by the integration of these recommendations, which were primarily generated by LGBTQ+ populations.

### Limitations

This study had several limitations. The term dictionaries we developed are specific to the data set we were using, which may impact the ability for them to be adapted to other data sets without alterations. Additionally, LGBTQ+ GoFundMe cancer campaigns were identified solely through self-disclosure. While this was an appropriate method for our aims and can assist in research aiming to analyze the social position and the ways that homophobia and transphobia may be functioning within online crowdfunding for cancer, it is not a suitable method for assessing the prevalence of LGBTQ+ populations who use online crowdfunding sites as not all LGBTQ+ individuals may choose to disclose their identity online. Further, it is unclear how generalizable such data and subsequent analyses would be to the cancer survivor population as demographic factors are not systematically and consistently available. However, it is highly likely that such data and analyses are representative of the portion of cancer survivors who report behavioral financial hardship and cost-coping behaviors as this data set contains all active cancer-related crowdfunding campaigns available on GoFundMe. Finally, members of the SAB were highly educated. This may have influenced the level of engagement, particularly for SAB members who participated in additional data coding meetings and manuscript authorship and may impact the generalizability of these methods for groups with lower educational attainment.

### Conclusions

Overall, our SAB was highly engaged throughout the entire study by metrics of attendance and participation at all 4 meetings. Integration of community-engaged and web-scraping methodologies resulted in a data set in which LGBTQ+ campaigns are able to be identified at 95% confidence. The methodological grounding and step-by-step methods outlined above provide a roadmap for future research in which technology-based methods are used for equity research. Our findings indicate high feasibility for integrating community-based methods with technology-based methods. In a time of research in which automation and big data are being used at an increasing rate, it is crucial to continue to center community-engaged equity-based methods in such research [39]. Doing so has the potential to produce more high-quality, unbiased research in hard-to-reach or historically underrepresented populations such as LGBTQ+ cancer survivors.

## Appendix

Multimedia Appendix 1 Word cloud of words and phrases from reflections on positionality from members of the Crowdfunding Cancer Costs LGBT Study (C3 LGBT) study team.

## Abbreviations

C3 LGBT: Crowdfunding Cancer Costs LGBT Study
LGBTQ+: lesbian, gay, bisexual, transgender, queer
SAB: study advisory board

## Data Availability

The data that support the findings of this study are available from the corresponding author, ARW, upon reasonable request.
