# Supplementary figures and images for: Exploring Online Crowdfunding for Cancer-Related Costs Among LGBTQ+ (Lesbian, Gay, Bisexual, Transgender, Queer, Plus) Cancer Survivors: Integration of Community-Engaged and Technology-Based Methodologies
Source: JMIR Cancer. 2023 Oct 30;9:e51605. doi: 10.2196/51605 (PMC10644187; doi:10.2196/51605)

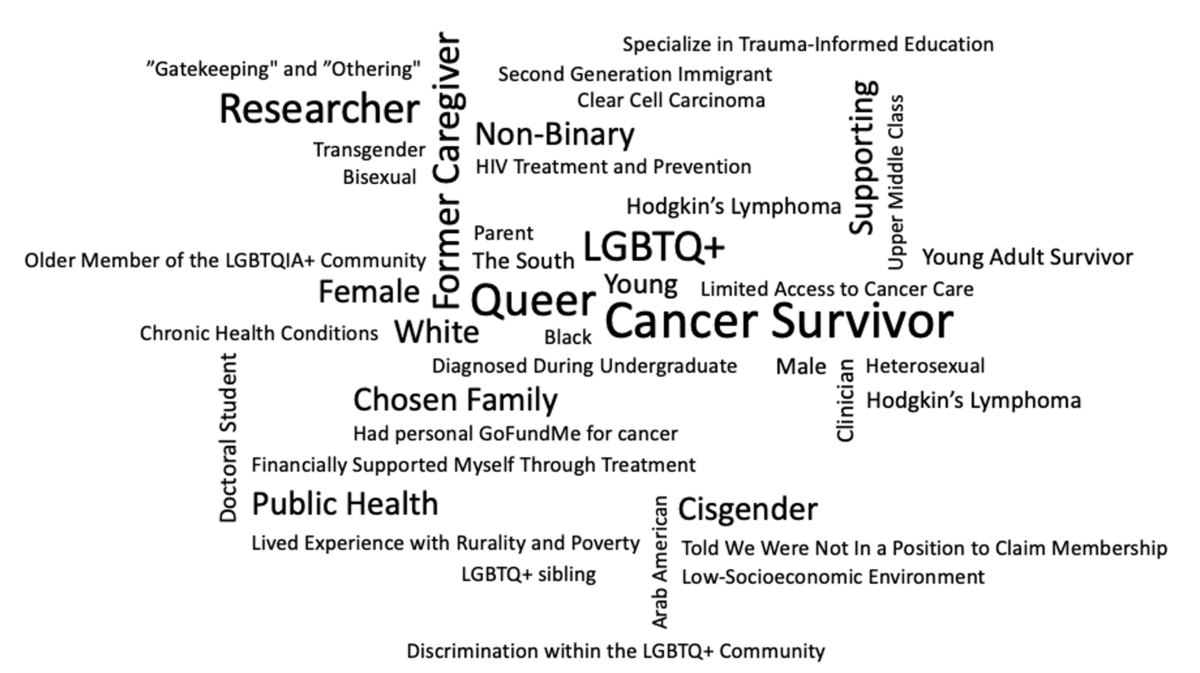

Supplement: Multimedia Appendix 1 [file cancer_v9i1e51605_app1.png]
